# Reconstruction of Chitosan Network Orders Using the Meniscus Splitting Method for Designing pH-Responsive Materials

**DOI:** 10.1021/acs.langmuir.4c00273

**Published:** 2024-05-31

**Authors:** Thi Kim Loc Nguyen, Yoshiya Tonomura, Nobuaki Ito, Ayaka Yamaji, Go Matsuba, Mitsuo Hara, Yuka Ikemoto, Kosuke Okeyoshi

**Affiliations:** † Graduate School of Advanced Science and Technology, Japan Advanced Institute of Science and Technology, 1-1 Asahidai, Nomi, Ishikawa 923-1292, Japan; ‡ Center for Nano Materials and Technology, Japan Advanced Institute of Science and Technology, 1-1 Asahidai, Nomi, Ishikawa 923-1292, Japan; § Graduate School of Organic Materials Science, Yamagata University, 4-3-16 Jonan, Yonezawa, Yamagata 992-8510, Japan; ∥ Department of Molecular and Macromolecular Chemistry, Graduate School of Engineering, Nagoya University, Furo-cho, Chikusa-ku, Nagoya 464-8603, Japan; ⊥ Japan Synchrotron Radiation Research Institute, 1-1-1, Kouto, Sayo-cho, Sayo-gun, Hyogo 679-5198, Japan

## Abstract

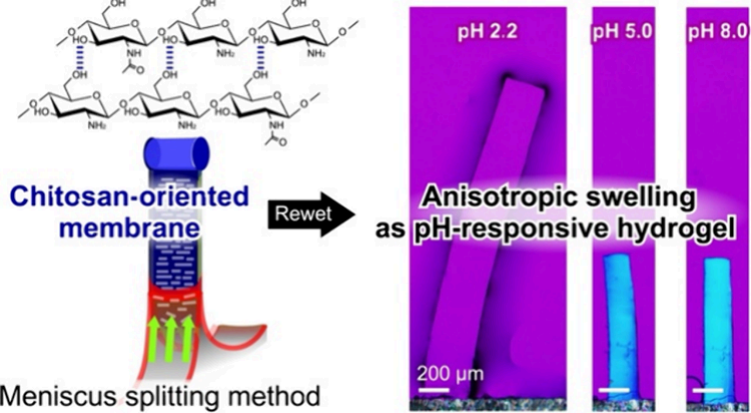

Chitosan is a product of deacetylated chitin and a natural polymer that is attractive as a functional and biocompatible material in the pursuit of alternative materials to synthetic plastics for a sustainable society. Although hierarchical architectures, from precise molecular structures to nanofibers and twisted structures, have been clarified, the expansion of the anisotropic microstructures of chitosan into millimeter-scale materials is in the process of development. In this study, a chitosan network was reconstructed from an aqueous solution by using the meniscus splitting method to form a three-dimensionally ordered microstructure. A chitosan membrane deposited on the millimeter scale formed a useful anisotropically pH-responsive hydrogel. During the evaporation of the aqueous solution from a finite space, chitosan underwent ordered deposition by capillary force to form a membrane with oriented microstructures and microlayers. Unlike the cast films formed between solid–liquid and air–liquid interfaces, this membrane formed between two air–liquid interfaces. As a result, the membranes with ordered microstructures were capable of signifying directional swelling in aqueous environments and reversible/irreversible swelling–deswelling changes by controlling the pH range. We envision that the anisotropic pH response of the chitosan network can be utilized under physiological conditions as a next-generation material.

## Introduction

As natural polymers, polysaccharides are expected to be alternative materials in the wide fields of food packaging, functional foods, drug delivery, cosmetics, agriculture, and other fields toward a sustainable society. In contrast to synthetic polymers, such as poly(vinyl alcohol) and polyacrylamide, natural polysaccharides, such as cellulose and chitosan, are widely researched as safe sources for human bodies and the global environment.^1−4^ Chitosan is a product of deacetylated chitin, which is extracted from the exoskeletons of crustaceans, insects, and fungal cell walls. Unlike most polysaccharides, chitosan is a cationic polymer with a positive charge that has attracted the attention of numerous researchers in various biomedical fields, such as wound healing, hemodialysis membranes, drug and gene delivery systems, implant coating, and tissue engineering/regeneration.^5,6^ In the side chain, amino and hydroxyl groups can be employed as cross-linkable groups. Amino groups can be protonated to ammonium groups below pH 6.4, rendering chitosan a pH-responsive soft material.^7,8^ However, hierarchically architectured microstructures of chitosan with anisotropy from bundles of nanofibers have not been utilized with pH-responsiveness. To use these self-assembled structures and their pH-responsiveness, a preparation methodology is required.

Recently, several methods, such as prestretching and drying,^9^ directional freezing,^10^ and electrospinning,^11^ have been reported for the preparation of polysaccharide-based materials with anisotropic microstructures.^12,13^ In contrast to these methods, we originally developed a meniscus splitting method^14−19^ for preparing a three-dimensionally ordered polymer membrane by developing viscous fingering phenomena.^20−23^ Under the control of the temperature and humidity, the evaporative interface of the polymer solution/dispersion from a cell induces the orientation of polymeric microfibers along the contact line of the interface by capillary force. During water evaporation, the concentrated polymer at the interface bridges the millimeter-scale gap between the two substrates and forms vertical membranes. The limitation for the bridging distance is basically ∼2 mm, depending on the capillary length.^23^ Notably, the dried membranes exhibit microstructures that are oriented parallel to the gap direction and layered microstructures in the thickness direction. These three-dimensionally ordered anisotropic architectures have been demonstrated in several types of polysaccharides, such as pectin, sacran, and xanthan.^14−19,24,25^

In this study, we reconstructed a chitosan network with three-dimensional order and designed a chitosan hydrogel that exhibited anisotropic swelling/deswelling with pH changes. The network was prepared in two steps: (I) an acidic aqueous solution of chitosan was dried using the meniscus splitting method to obtain microscopically oriented and layered structures in a dried state and (II) the dried chitosan membrane was immersed in pH buffer solutions to control the intermolecular cross-linking points in the membrane. Considering that pH control is an important strategy in the field of biomedicine, sol–gel transitions via pH changes in aqueous solutions should be clarified. To confirm the composition/decomposition of the cross-linking points, the pH dependence of the hydrogels was studied in three dimensions. Using the tendency that intermolecular hydrogen bonding among the hydroxy groups in the side chain works as cross-linking points,^26−28^ the oriented structures of chitosan fibers in the dried membrane were maintained during in situ cross-linking. Based on the membrane preparation with orientation control, the microstructures reflected anisotropic swelling/deswelling as pH-sensitive hydrogels. To clarify the anisotropic nano- and microstructures in the dried membranes, we conducted microscopic observations using polarized optical microscopy, scanning electron microscopy (SEM), wide- and small-angle X-ray scattering (WAXS, and SAXS), and polarized attenuated total reflection infrared spectroscopy.

## Experimental Section

### Materials

Chitosan, as poly(d-glucosamine) with a *M*_w_ of 50–190 kDa, was purchased from Merck KGaA, Darmstadt, Germany. The product was 75–85% deacetylated, and the viscosity of the aqueous solution was 200–800 cP (1 wt % in 1% acetic acid). This is the deacetylated chitin, poly(d-glucosamine), which is categorized as a low molecular weight. Acetic acid (99.7%, FUJIFILM Wako Pure Chemical Corp., Osaka, Japan) was used as received. After the chitosan powder was dissolved in an aqueous solution containing acetic acid, a small amount of impurities and air bubbles were removed using centrifugation using a centrifuge (CF15RN, Eppendorf Himac Technologies Co., Ltd., Japan) and an angle rotor (Angle Rotor T15A36, Eppendorf Himac Technologies Co., Ltd., Japan), under conditions (21,800 × *g*, 25 °C, 30 min). McIlvaine buffer solutions composed of citric acid (Merck KGaA, Darmstadt, Germany) and disodium phosphate (Kanto Chemical, Tokyo, Japan) were prepared by using chemicals without purification.

### Drying Experiments

The aqueous solution at ∼25 °C was poured into a topside open cell, which is a type of Hele-Shaw cell. The cells were placed in an oven at constant temperature under atmospheric pressure using the air circulator of a forced convection system (AS ONE, OFWP-600 V). Considering that the volume of the oven (600 mm × 497 mm × 500 mm, ∼150 L) with an air circulator was larger than the volume of the samples (<1 mL), the RH in the oven was controlled by the set temperature.

### Characterizations

Wide-angle X-ray scattering (WAXS) measurements were performed with a nanoviewer (Rigaku Co., Tokyo, Japan) using a Cu–Ka radiation source. The wavelength of the X-ray beam used was 0.154 nm, and the scattered X-rays were detected by a 2D detector, PILATUS 1 M (Detris A.G., Baden, Switzerland). Small-angle X-ray scattering (SAXS) measurements were conducted using an FR-E instrument equipped with a two-dimensional detector, R-axis IV (Rigaku), and an imaging plate (Fujifilm). An X-ray beam from Cu Kα radiation 0.154 nm, 0.3 mm collimated) was used, and the camera length was set to 300 mm. The samples were packed in glass capillary tubes (Hilgenberg) with a diameter of 1.0 mm and a thickness of 0.01 mm and were subsequently measured in an open system, in a dry state. After measurement in the dry state, pure water was loaded into the capillaries, and 10 min later, the samples were measured in a wet state. IR spectra (in the mid-IR region from 800 to 4000 cm^–1^) were obtained using the IR beamline BL43IR at the SPring-8 synchrotron facility (Hyogo, Japan). An FTIR microspectrometer (Bruker model Hyperion2000 IR microscope with a Vertex 70 FTIR spectrometer) was used with IR synchrotron radiation. The wavenumber resolution was 3 cm^–1^, and the accumulated number was 200. Attenuated total reflectance (ATR) spectra were obtained by attaching the ATR objective (20× and NA = 0.6 objective with Ge ATR crystal) onto the Hyperion2000 microscope. Polarized spectra were obtained by using a BaF_2_ substrate wire-grid polarizer.

### Microscopic Observations

To verify the macroscopic orientation, the samples were photographed using linear-crossed polarizers. To determine the orientation direction, a first-order retardation plate with λ = 530 nm was placed in the light path between a polarizer and the sample. Microscopic observations were performed by using a microscope (BX53-P, Olympus) equipped with a CCD camera (DP74, Olympus). The swelling behavior of the hydrogels in terms of *L*_*x*_, *L*_*y*_, and *L*_*z*_ was monitored using a microscope, and the thickness (*L*_*x*_) was checked using a micrometer to ensure the reliability of the measurements. To characterize the submicrometer-scale structures of the membranes, the samples were observed using field-emission SEM (S-5200, Hitachi). The samples were coated with an ion sputterer (Neoc-Pro, Meiwafosis Co., Ltd., Japan).

## Results and Discussion

**Figure 1:**
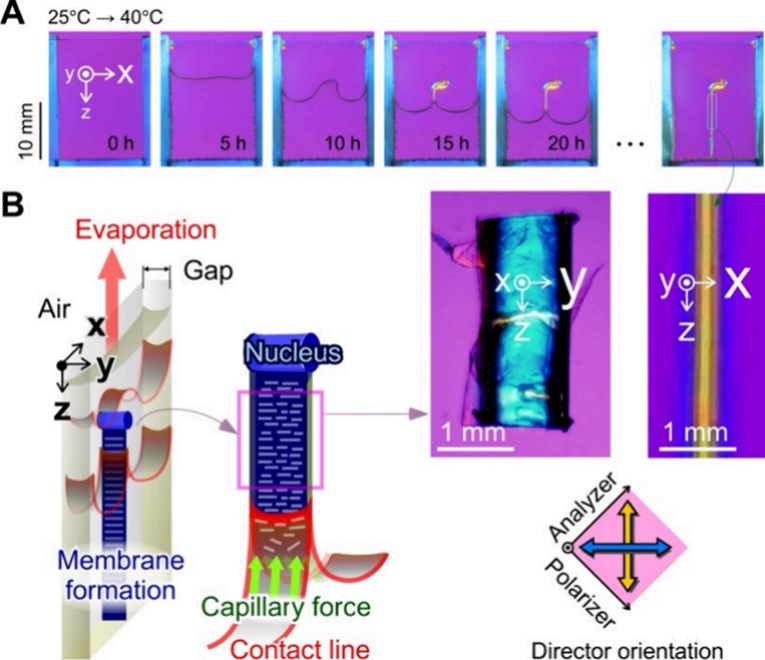
Preparation of chitosan membranes using the meniscus splitting method. (A) Time course change of chitosan solution from a Hele-Shaw cell (15 mm-width, 1 mm-gap, ∼20 mm-depth) observed through crossed Nicols with a retardation plate (λ = 530 nm). (B) Polarized microscopic images show the dried membrane in the *YZ*-plane and *XZ*-plane. Director orientation is common for all images.

The meniscus splitting method enabled the aqueous chitosan solution to form a membrane (Figure 1). Chitosan was initially dispersed in an aqueous mixture containing acetic acid and deposited by bridging the gaps and forming a membrane during drying. Acetic acid was used to disperse chitosan that was not dispersed in pure water (chitosan: 2.5 wt %, acetic acid: 1 v/v%). The chitosan solution was poured into a Hele-Shaw cell with a 1 mm gap. The gap size for the polymer bridging and the membrane formation is optimized, based on the principle of capillary action^23^ and experimental demonstrations (see Supporting Information, Figure S1). In the condition, the probability of the meniscus splitting for the membrane formation was high enough, >90%, and reproducible. The membrane having the orientation parallel to the *Y*-direction was confirmed in range of 0.5–2.0 mm. During this process, the solutions were heated at 40 °C, where both water and acetic acid were evaporated (Figure 1A, and Movie S1). The heating temperature at 40 °C stably provides membrane formation with the layered structure (Supporting Information, Figure S2). The liquid phase showed pink color and the same color in the background and did not show blue or yellow color, suggesting that it is the isotropic state in this scale. Next, polarized light microscopic observation of the membrane revealed that the polarizers and first order retardation plate (λ = 530 nm) were directionally set, as shown Figure 1B. Unlike cast films on the bottom or sidewalls with no signals of orientation (Supporting Information, Figure S3), the membrane formed from two air–liquid phases, which is shown in blue. This result strongly suggests that the membrane has a structure that is oriented parallel to the Y-gap axis. This is because the polymer orients along the contact line in the *Y*-gap direction, where the capillary force works strongly, similar to other polysaccharides previously reported.^14,17^ The polarized microscopic image in the *XZ*-plane showed a strong yellow collar, suggesting that the polymer aligns parallel to the *Z*-direction.

**Figure 2:**
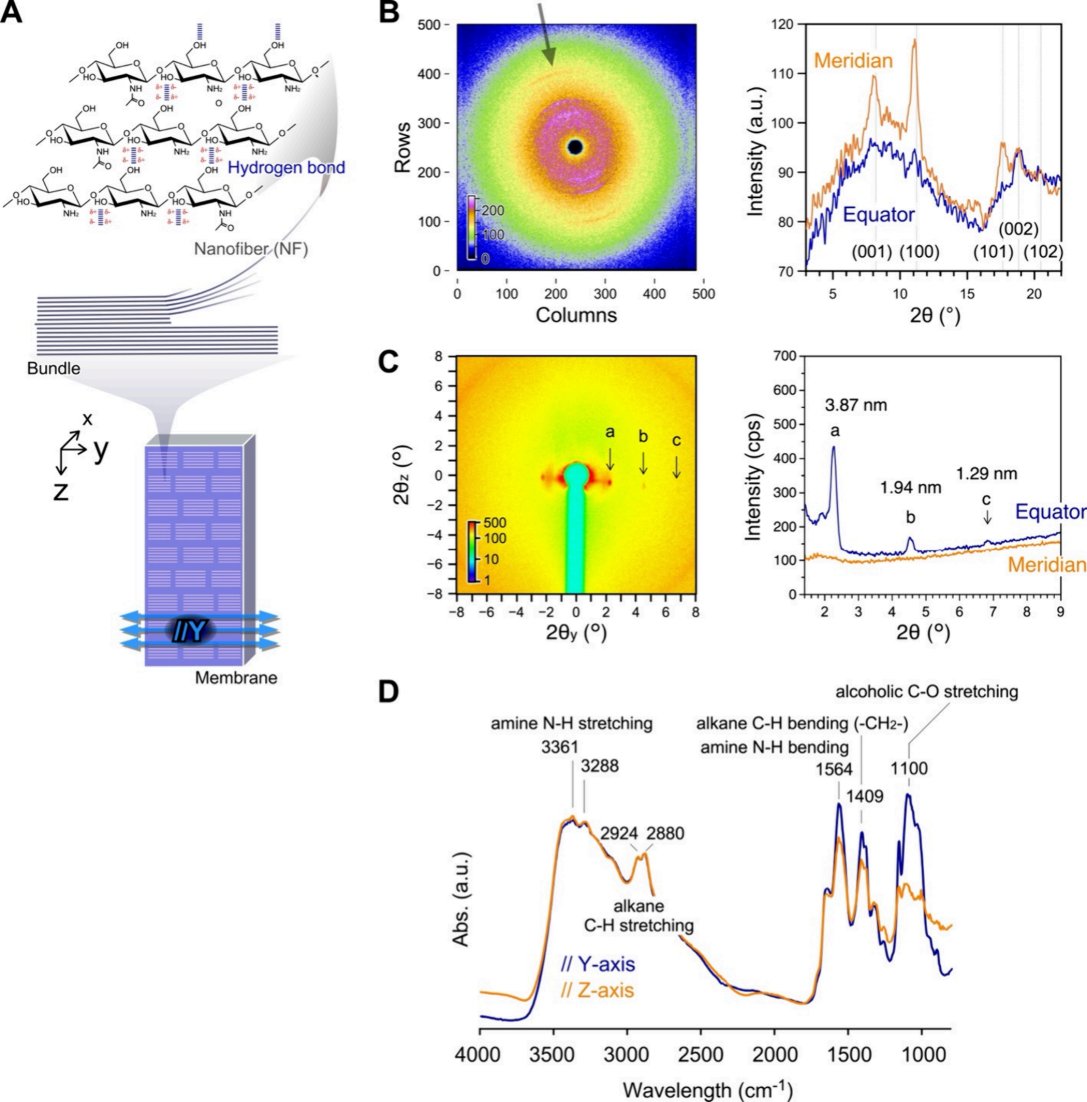
Nano- and molecular structures in the chitosan dry membrane prepared using the meniscus splitting method. (A) Schematic illustration of the chitosan membrane prepared using the meniscus splitting method and its chemical structure. *Y*-axis is the gap direction, and the *Z*-axis is the depth direction in the cell. The XY*Z*-axis is common with the preparation using meniscus splitting from Hele-Shaw cells. (B) WAXS profiles of the membrane in an air environment. The arrow indicates the position of strong intensity. (C) SAXS profiles of dry membrane under an air environment. The arrow indicates the position of strong intensity. Room temperature: ∼25 °C. Relative humidity: ∼50%. (D) IR spectra of the membrane under polarized light. Spectra in blue and yellow are detected when the polarizer is parallel to the cell gap (*Y*-direction) and cell depth directions (*Z*-direction), respectively. Room temperature: ∼ 25 °C. Relative humidity: ∼50%.

To clarify the nano- and molecular structures of the chitosan membrane, samples were analyzed using WAXS and SAXS. Figure 2A shows a schematic illustration of the membrane as a dried sample and its chemical structure with hydrogen bonds as the intermolecular interaction. The membrane has a hierarchical architecture: nanofibers and microfibers as the molecular and nanofiber bundles, respectively, as in previous reports.^29−31^ As shown in Figure 2B, the WAXS profile indicates that characteristic periodicities exist on the nanometer scale. The profile was calibrated, centered, and integrated along the meridional and equatorial directions. The equatorial peaks indicate the distances along the intersheet and interchain directions. The arrow in the 2D WAXS profile was analyzed as the 1D WAXS profile, showing the orientation functions (001), (100), and (101) in the meridian but not in the equator. This result strongly indicates that the fibrillar crystals were detected in the meridian direction. In addition, this signal was a marker of the distance between the two hydroxyl groups along the central axis of the chitosan helix. Figure 2C shows SAXS profiles of the chitosan membrane measured in air. The chitosan membrane exhibited a large peak at 2θ ≈ 2.2° and two smaller intense peaks at 2θ = 4–6°, which correspond to the periodicity of 3.87 and 1.94–1.24 nm in the equatorial direction, respectively. These periodicities would be originated from the diameter of chitosan nanofibers. In addition, these suggest the presence of anisotropic lamellar stacking structure oriented parallel to the *Z*-direction. In contrast, the peaks observed in air disappeared in the pure water environment (Figure S4). This could be due to plenty of water molecules penetrating the periodic structures, thereby resulting in anisotropic swelling.

To clarify the anisotropy of the molecular structures, the dried sample was evaluated using infrared (IR) spectroscopy through polarized light at 50% relative humidity (RH) (Figure 2D). The two significant peaks at approximately 3361 and 3288 cm^–1^ were attributed to the O–H and N–H stretching vibrations, respectively, of the functional groups, which are involved in hydrogen bonding between chitosan molecules. The peaks at 2880 and 2924 cm^–1^ indicated alkane C–H stretching. The characteristic peaks at 1564, 1409, and 1100 cm^–1^ are attributed to the N–H bending vibration of amine II, alcoholic C–O stretching, and alkane C–H bending, respectively. All bands, except those at 3288, 3361, and 2880–2924 cm^–1^, in the direction parallel to the *Y*-axis were stronger than those in the direction parallel to the *Z*-axis. These results can be attributed to the fact that the chitosan nanofibers in the bundled state exposed more side chains parallel to the *Y*-axis.

**Figure 3:**
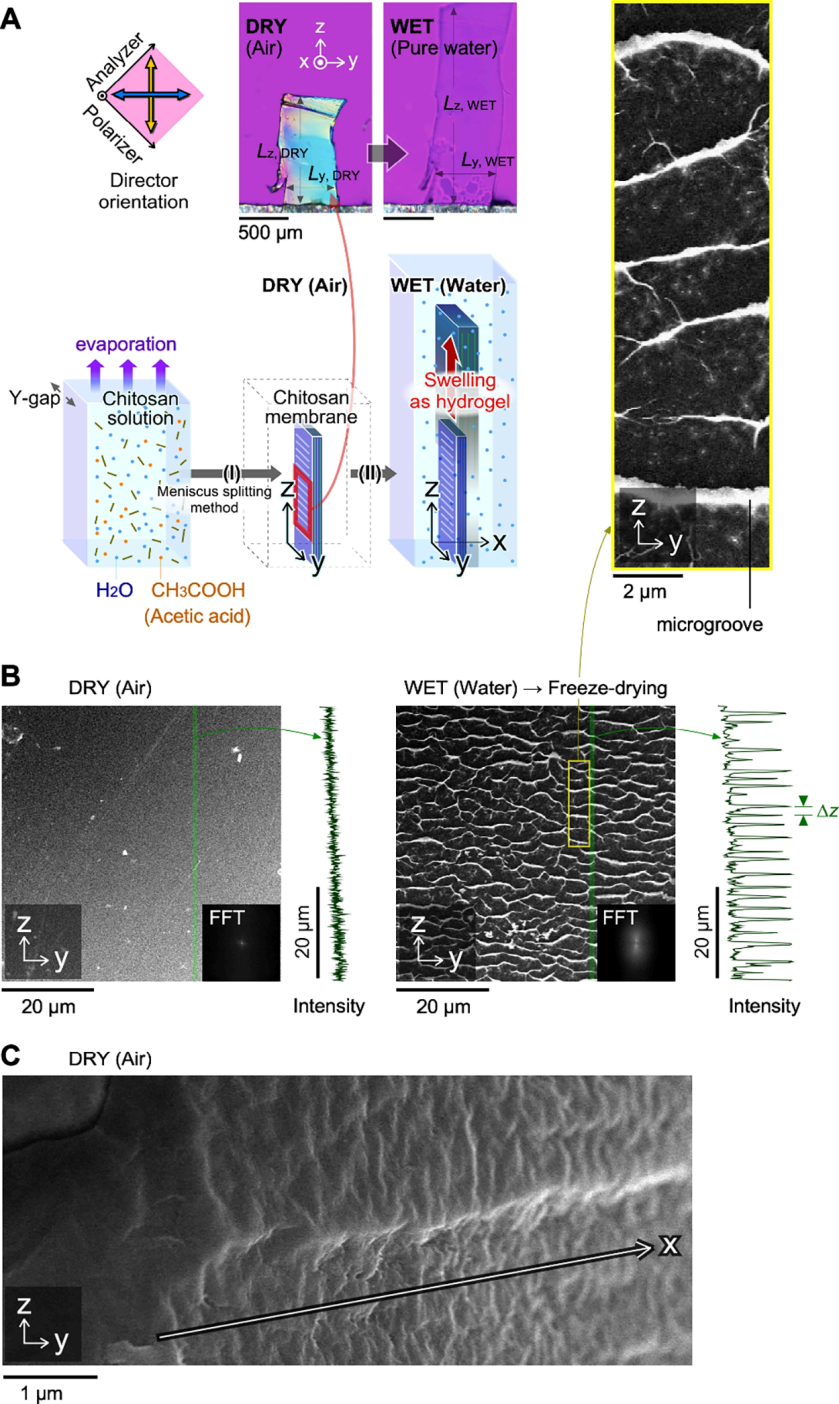
Chitosan hydrogel anisotropically swelling in pure water and its microstructures. (A) Polarized optical microscopic images in air and in pure water. The process of the chitosan solution for the preparation of the DRY membrane and WET hydrogel. (I) Drying the aqueous chitosan solution includes acetic acid to form the chitosan membrane using meniscus splitting. (II) Addition of pure water to the membrane. (B) SEM images of the heat-dried sample prepared using the meniscus splitting method as DRY (air) and the freeze-dried sample as WET (water). Inset: fast Fourier transformation (FFT) analytical data of SEM images. Charts indicate surface asperity on the YZ-plane of samples by analyzing the intensity of the gray value along the green line in the SEM images. Zoomed-in image in yellow-outlined area shows typical periodicity. (C) SEM images of the heat-dried sample, focusing on the cross-section in the *X*-direction.

Considering that chitosan cannot disperse in pure water but can disperse in an acetic acid aqueous solution, the dried chitosan membrane with an oriented structure should have the potential to show ordered swelling. The thickness of the dried membrane was in range of 90–110 μm. The uniformity of the thickness could be confirmed in parts of 10 mm height in the middle (see Supporting Information, Figure S1). By rewetting the membrane with pure water, and without any additional cross-linker from air, the membrane behaved as a hydrogel with anisotropic swelling (Figure 3A and Supporting Information, Movie S2). The swelling ratios, *L*_WET_/*L*_DRY_ in the *X*-, *Y*- and *Z*-directions were 3.1, 1.3, and 1.8, respectively. During drying of the chitosan solution, chemical cross-linkers or cross-linking points were not purposely introduced. Notably, the membrane rapidly and anisotropically swelled in pure water while maintaining its hydrogel shape. This behavior suggests that the oriented structure in the *Y*-gap direction affected the swelling direction. Certain hydroxyl groups on the chitosan side chains can function as cross-linking points for hydrogen bonds to behave as a hydrogel. During anisotropic swelling from the dry state in air to the wet state in pure water, the intensity of the blue color decreased, but the sample retained its size in the *Y*-direction. These results clearly indicate that numerous water molecules can anisotropically interpenetrate in the striped structures along the *Y*-direction but not along the *Z*-direction. As a result, the membrane remarkably swelled in the *Z*-direction.

To study the microstructures in the DRY/WET states, the samples were observed by using SEM (Figure 3B). A WET (water) sample was prepared by freeze-drying. In contrast to the DRY sample, the WET sample had striped patterns with microgrooves parallel to the *Y*-axis. This tendency was also confirmed with analytical data from FFT (Figure 3B, inset). Microgrooves were generated during the sample preparation under reduced pressure, enabling mechanical stress. By analyzing the gray values of the green line on the images, the interval sizes between the microgrooves (Δ*z*) were measured. The average value of Δ*z* was 2.1 μm, and the groove width was <0.3 μm. The striped shape with values clearly signifies that the periodic microstructures in the *Z*-direction were maintained in the membrane. Such submicrometer-scale periodicity was also confirmed in the cross section of the DRY membrane as layers of the *YZ* plane stacked in the *X*-direction (Figure 3C and Supporting Information, Figure S5). Considering that the layer-to-layer distance varied 0.3–0.5 μm, the chitosan fibers were easily self-assembled at this submicrometer-scale, as well as the chitin.^31^ Conversely, the twisted plywood structures typically observed in chitin were not clearly observed, and the chitosan membranes may contain twisted structures. This consideration is based on the fact that the membrane has two strong, two-oriented structures parallel to the *Y*- and *Z*-axes.

**Figure 4:**
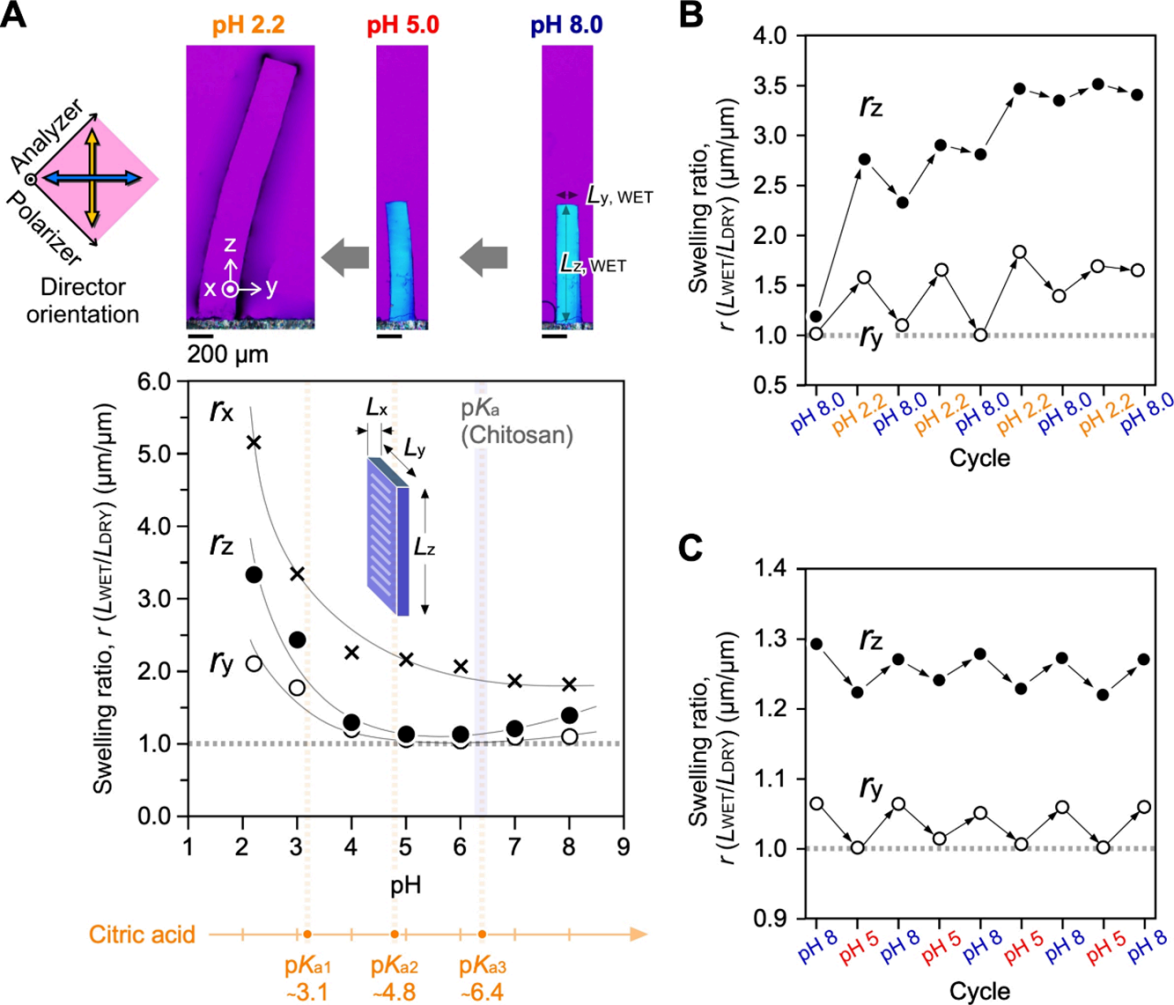
Chitosan hydrogel with an anisotropic pH response. (A) pH-dependence of the swelling ratio in three dimensions. *L*_*x*_, *L*_*y*_, and *L*_*z*_ represent the sample lengths in each direction. Swelling ratio in each direction, *r* indicates the *L*_WET_/*L*_DRY_. For example, *r*_*x*_ = *L*_WET, x_/*L*_DRY, and x_. DRY signifies a condition under air atmosphere at approximately 25 °C. The DRY samples were equilibrated in initial pH buffer solutions as the WET state. (B) Repeat tests of pH changes between 8.0 and 2.2. (C) Repeat tests of pH changes between pH 8.0 and 5.0. Buffer solutions were composed of citric acid and disodium phosphate.

To three-dimensionally understand anisotropic swelling, the dried membrane was directly immersed into each buffered solution with a wide pH range (2.2–8.0) at ∼25 °C. Figure 4A shows typical images at a given pH and the pH dependence of the equilibrium swelling ratio. The ratio, *r*, is defined as the ratio of the length in the wet state to that in the dry state, *L*_WET_/*L*_DRY_. Swelling ratios in each direction are defined as *r*_*x*_ = *L*_WET, x_/*L*_DRY, or x_, for example. In this pH range, *r*_*x*_ was significantly higher than those of *r*_*y*_ and *r*_*z*_. For example, *r*_*x*_ at pH 5.0 was 2.1 (*L*_WET, x_ = 72 μm, *L*_DRY, x_ = 34 μm). This remarkable *r*_*x*_ increase might be due to the greater change in the intervals between the layers, as confirmed by the SEM images (see Figure S5). All the swelling ratios (*r*_*x*_, *r*_*y*_, and *r*_*z*_) in acidic conditions with pH 2.2–4.0 showed higher values, and the polarized microscopic images showed no signals for orientation in the *Y*-direction. Under acidic conditions, protonation enables water molecules to penetrate the interspaces among the fibers and chains in the fibers. Consequently, certain cross-linking points, owing to hydrogen bonds, can be decomposed. Considering that the p*K*a of chitosan is approximately 6.4, the protonation/deprotonation equilibrium condition affects the swelling ratio in the pH range of 5.0–7.0.

The repeatability of swelling/deswelling as a hydrogel was confirmed for the use of the membrane under physiological pH conditions, such as a pH of 7.4 in blood. In the cyclic pH changes between 8.0 and 2.2, the gels showed no reversibility. After swelling from pH 8.0 to 2.2, the gel once again immersed at a pH of 8.0 did not deswell to its original ratio and maintained a similar ratio to that at pH 2.2 (Figure 4B). Following the repeated tests of pH-changes between pH 8.0 and 2.2, certain cross-linking points irreversibly decomposed at a pH of 2.2, and the sample continued swelling close to the equilibrium state of pH 8. This irreversible tendency was also confirmed in the pH cyclic changes between 5.0 and 2.2. Under acidic conditions, such as a pH of 2.2, protonation of the chitosan chain strongly induced the decomposition of hydrogen bonds between the hydroxy groups and an exchange of citric acid. Notably, in the cyclic pH changes between pH 8.0 and 5.0, the chitosan gels exhibited reversibility both in the *Y*- and *Z*-directions (Figure 4C, Supporting Information Table S1 and Figure S6). In contrast to the *r*_*y*_ changes (1.0 and 1.1), the *r*_*z*_ changes were approximately 1.2 and 1.3. The small increases of the *r*_*z*_ as the pH change from 5 to 8 would be because that the decrease of citric acid concentration and the increase of the Na_2_HPO_4_ in the buffer. The increase of PO_4_^3–^ concentration would intervene in the intermolecular complex between the hydroxy groups in the pH 8.0 condition. These results suggest that water molecules can penetrate in and out of the intervals of the oriented main chitosan chains but some of the hydrogen bonds remain without dissociation. This reversibility of the anisotropic swelling is due to the rigid chains parallel to the *Y*-direction. This consideration is supported by the data for structural analyses at the nano- and molecular scales (Figure 2A–D).

**Figure 5:**
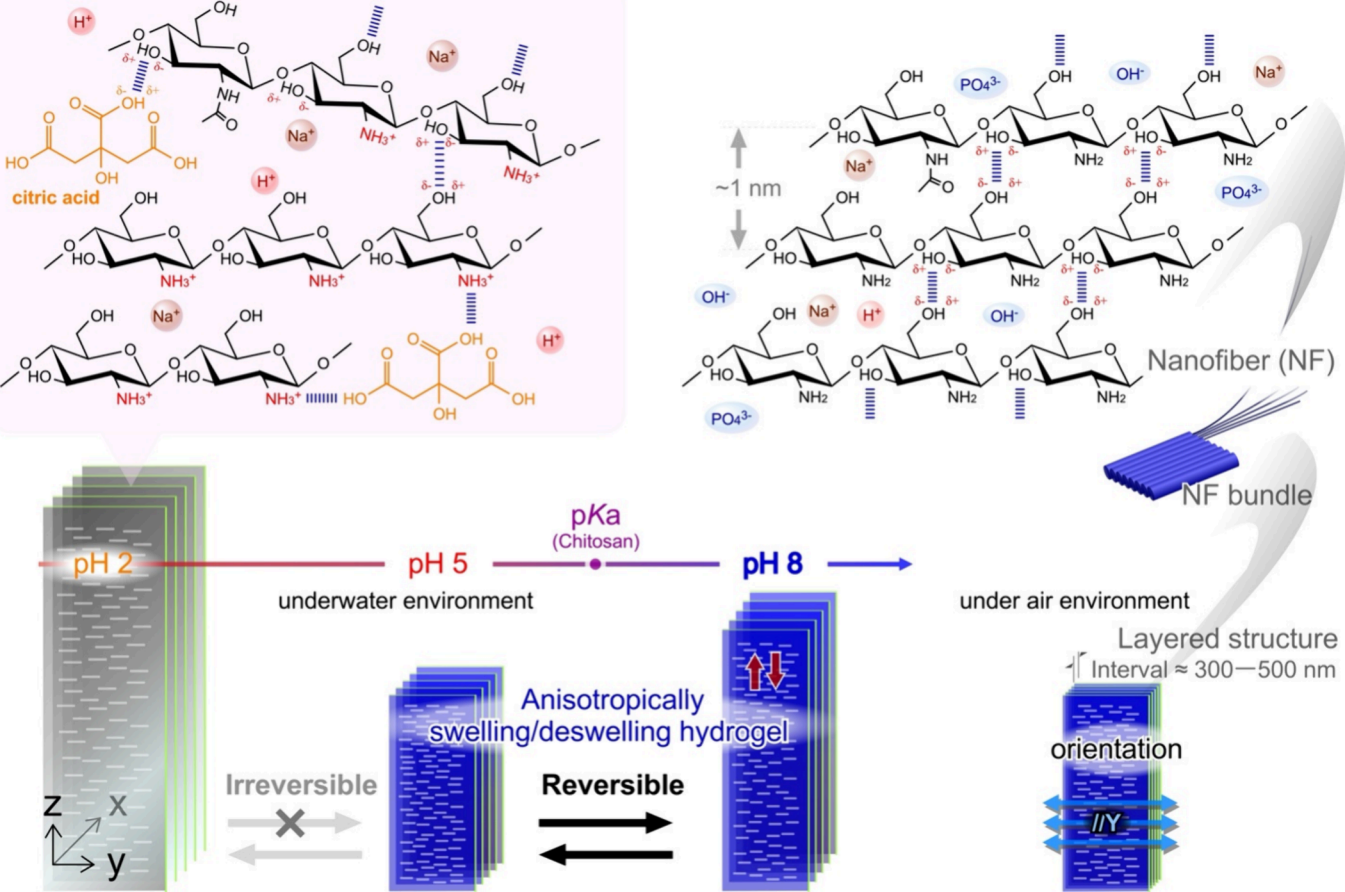
Schematic illustration of the chitosan membrane functioning as a pH-responsive hydrogel with anisotropy. Chitosan hydrogel prepared using meniscus splitting shows reversible swelling and deswelling between pH 5 and 8. It shows an irreversible swollen state following the repetition of exchanging outer buffer solution between pH 2.2 and 8. pH was controlled by citric acid and disodium phosphate concentrations.

Figure 5 summarizes the structures of the chitosan membranes at multiple scales prepared by using the meniscus splitting method and the anisotropic swelling/deswelling behaviors of the hydrogel in buffered solutions. Considering that the membrane was prepared by drying an aqueous chitosan solution containing concentrated acetic acid, the chitosan chains or crystalline structures could accumulate from the dispersed state to the oriented state on the evaporative interface. This orientation process, driven by capillary force, is similar to that of other polysaccharides with self-assembled microfibers.^14−19^ Dried chitosan membranes have an oriented structure with molecular, nanometer, and submicrometer structures. Hierarchical architecture is the basic skeleton for the in situ cross-linking and anisotropic swelling of hydrogels. Regarding the reversibility of swelling/deswelling, the cross-linking points in the chitosan membranes were primarily affected by the concentration of citric acid in the buffer solutions. The acid dissociation constant (p*K*a) for chitosan is approximately 6.4, and those for citric acid with three carboxylic acid groups are approximately 3.1, 4.8, and 6.4. The pH condition also affects the increase in the protonated amino groups that induce the increase in the osmotic pressure in the network. Based on the correlation between the protonated or deprotonated states of chitosan and citric acid, the swelling ratios were affected by the formation of electrostatic attraction between the protonated amino groups of chitosan and the deprotonated carboxyl groups of citric acid. Thus, it exhibited an irreversibly swollen state because most of the hydrogen bonds among the chitosan chains were exchanged with acids.

## Conclusions

A chitosan membrane with a three-dimensionally ordered structure was successfully prepared by using the meniscus splitting method. This membrane was capable of functioning as a hydrogel with an anisotropic pH response over a wide pH range of 2.2–8.0. Analysis of the dried membrane using WAXS and SAXS confirmed that the chitosan chains reconstructed the orientation in the molecular and nanostructures. In particular, the clear difference in the IR spectra between the observation directions (//*Y* or //*Z*) indicates that the millimeter-scale membrane exposes side chains, such as −NH_2_ and – OH, that are parallel to the gap direction (//*Y*) more than the depth direction (//*Z*). This reconstruction of anisotropy from the aqueous solution was also confirmed by using polarized optical microscopy and SEM, indicating a macroscopic orientation in the membrane. This anisotropic structure directly affected the anisotropic swelling of the hydrogel in aqueous environments. Through in situ intermolecular cross-linking via hydrogen bonding among the hydroxyl groups on the side chain, the swelling ratio was three-dimensionally controlled under acidic to alkaline conditions. The ratio was strongly affected by acidic conditions. One is the increase in the osmotic pressure as an increase in the protonated amino groups arranged along the direction parallel to the gap direction (//*Y*). The other is caused by intermolecular cross-linking with intermediate citric acids. Based on the pH-responsive swelling ratio, this material can be applied in an aqueous environment with a pH signal, irreversible in the pH range 2–5 and reversible in the pH range of 5–8. The decrease in pH from normal cells to cancer cells can be a switching signal not only for mass transporters, such as nanogels and micelles, but also for tissue engineering using stimuli-responsive soft materials. The anisotropic pH response of the chitosan networks under physiological conditions would be useful in wound dressing materials or materials for skin cell renewal.

## Supporting Information Available

Methods and experimental details (PDF)
Preparation of chitosan membranes using the meniscus splitting method (Movie S1) (MOV)
Swelling processes of membranes from the dry state in air to the wet state in pure water (Movie S2) (MOV)

## Supplementary Material

la4c00273_si_001.pdf

la4c00273_si_002.mov

la4c00273_si_003.mov
