# Supplementary material for: Reconstruction of Chitosan Network Orders Using the Meniscus Splitting Method for Designing pH-Responsive Materials
Source: Langmuir. 2024 May 31;40(23):11927–35. doi: 10.1021/acs.langmuir.4c00273 (PMC11171445; doi:10.1021/acs.langmuir.4c00273)
Supplement: Supplementary file 1 — la4c00273_si_001.pdf [file la4c00273_si_001.pdf]

Supporting Information

Reconstruction of chitosan network orders using the meniscus splitting method for designing pH-responsive materials

Thi Kim Loc Nguyen[1], Yoshiya Tonomura[1], Nobuaki Ito[2], Ayaka Yamaji[3], Go Matsuba[3], Mitsuo Hara[4], Yuka Ikemoto[5], Kosuke Okeyoshi[1]*

[1]Graduate School of Advanced Science and Technology, Japan Advanced Institute of Science and Technology, 1-1 Asahidai, Nomi, Ishikawa 923-1292, Japan

[2]Center for Nano Materials and Technology, Japan Advanced Institute of Science and Technology, 1-1 Asahidai, Nomi, Ishikawa 923-1292, Japan

[3]Graduate School of Organic Materials Science, Yamagata University, 4-3-16 Jonan, Yonezawa, Yamagata 992-8510, Japan

[4]Department of Molecular and Macromolecular Chemistry, Graduate School of Engineering, Nagoya University, Furo-cho, Chikusa-ku, Nagoya 464-8603, Japan

[5]Japan Synchrotron Radiation Research Institute, 1-1-1, Kouto, Sayo-cho, Sayo-gun, Hyogo 679-5198, Japan

*To whom correspondence should be addressed.
E-mail: okeyoshi@jaist.ac.jp

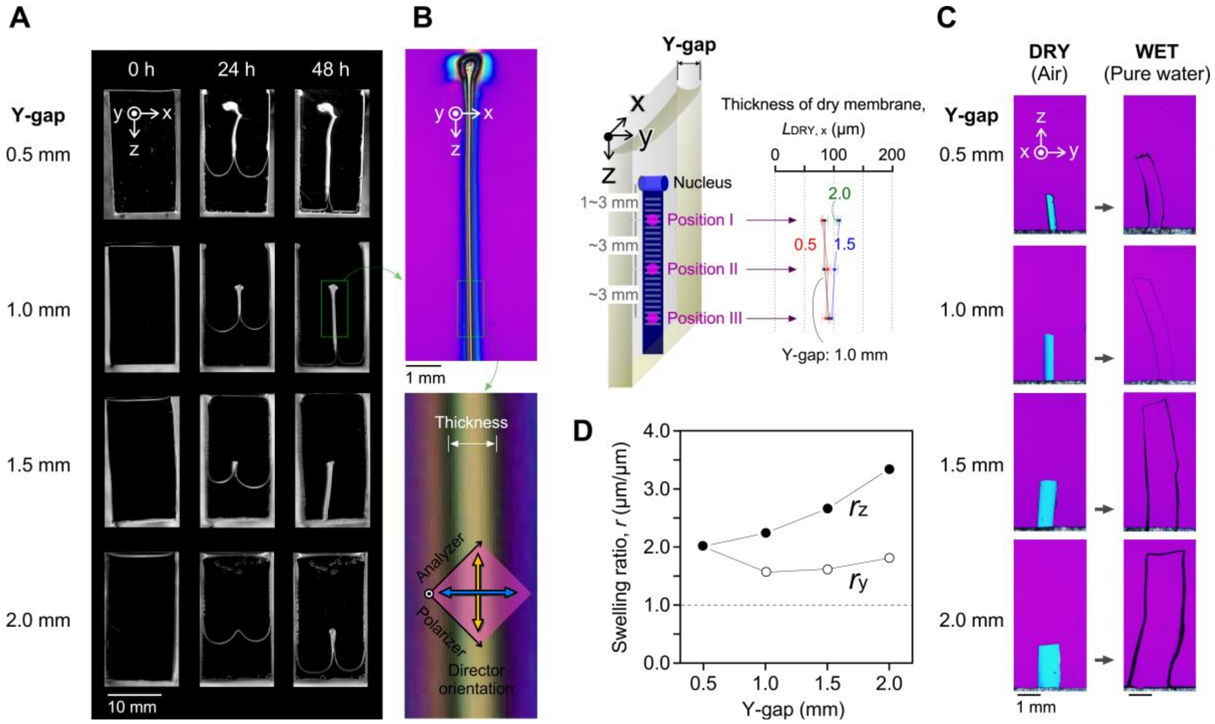

Figure S1. Chitosan membrane formation and polarized microscopic images by meniscus splitting method with controlling the cell gap. A. Membrane formation from a Hele-Shaw cell (12 mm, Y-gap, ~20 mm) with given gap distance (0.5, 1.0, 1.5, and 2.0 mm). Initial chitosan concentration: 2.5 wt%. Initial concentration of acetic acid: 1 v/v%. Drying temperature: 40˚C. **B.** Polarized optical microscopic images of membranes in XZ-plane and thickness of dried membrane, $L_{DRY,\,x}$ at given positions (I, II and III) in each gap-condition. **C.** Polarized optical microscopic images of membranes in YZ-plane under air and under pure water. All samples in B and C were observed through crossed Nicols with a retardation plate (530 nm) in the same direction as represented. **D.** Swelling ratio, $r = L_{WET}/L_{DRY}$ in pure water at ~25℃. Subscript in r means each direction (Y, and Z). For example, $r_y = L_{WET,\,y}/L_{DRY,\,y}$. L_y means the length of sample in the Y-direction. The axes are common for all panels.

The thickness of the dried membrane at given positions, less than 10 mm from the nucleus-top showed similar values in each gap-condition (**Fig. S1A-B**). By using the membranes from cell with 0.5, 1.0, and 1.5 mm-gap, the swelling ratio in pure water was measured. The value for 1.0 mm-gap was in range of ±20% similar with the values for 0.5 and 1.5 mm. It would be estimated that the membrane formed from a cell with 1.0 mm-gap would reproducibly show similar ratios (**Fig. S1C-D**).

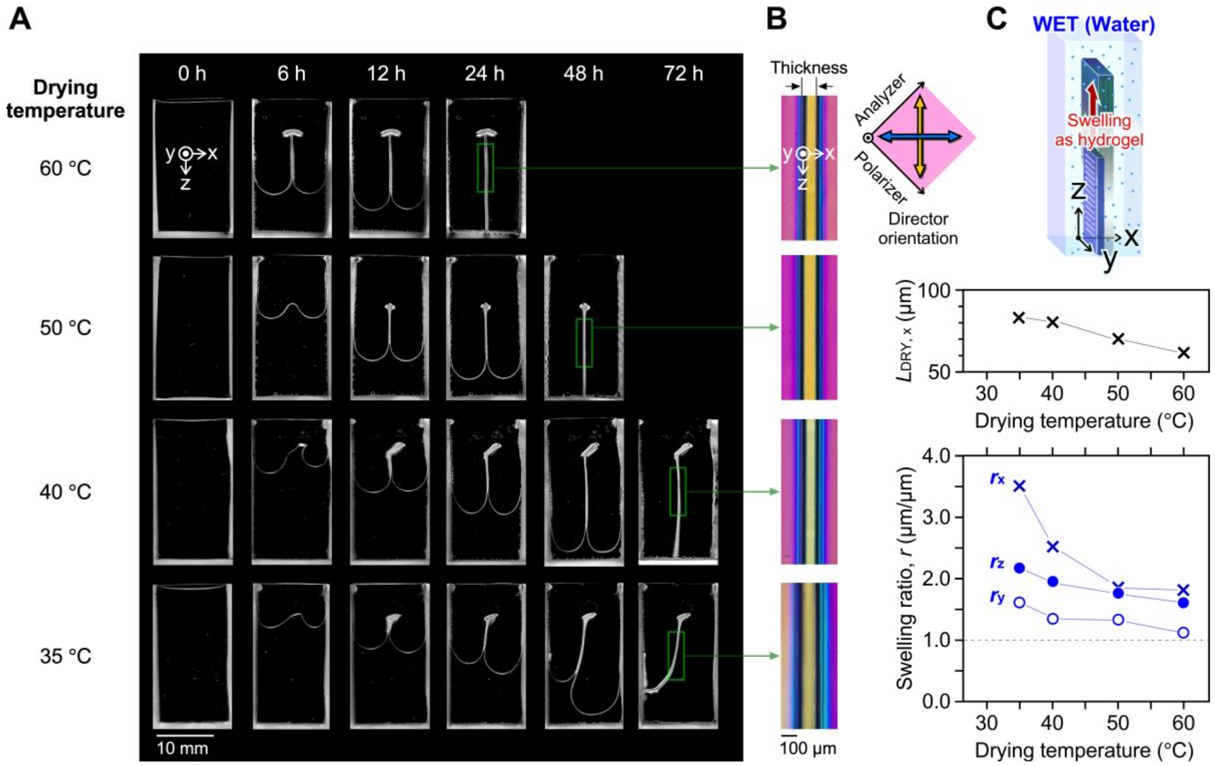

Figure S2. Effect of drying temperature on membrane formation through meniscus splitting. A. Photograph of deposited vertical membrane by drying chitosan solution. Initial concentration of chitosan: 2.5 wt%. Initial concentration of acetic acid: 1 v/v%. Drying from a Hele-Shaw cell (12 mm-width, 1 mm-gap, ~20 mm-depth). Drying temperature: 35, 40, 50, and 60 ˚C. **B.** Polarized optical microscopic images of membranes under air in XZ-plane through crossed Nicols with a retardation plate (λ = 530 nm). **C.** Thickness of dried membrane, $L_{\mathrm{DRY,\,x}}$ at given positions and swelling ratio in pure water in each direction as a function of drying temperature.

By checking the effect of the drying temperature on the membrane formation through meniscus splitting, the layered structure was qualitatively confirmed (**Fig. S2A**). In the drying temperature range of 35—60 ℃, the polymeric orientation could be suggested by the polarized microscopic observation (**Fig. S2B**). To verify the layered structures for absorbing water, the membrane's thickness ($L_{\mathrm{DRY,\,x}}$) and the swelling ration in each direction were measured (**Fig. S2C**). As an increase of the drying temperature from 35 ℃ to 60 ℃, the $L_{\mathrm{DRY,\,x}}$ decreased to ~70%. This trend is also sawn in the swelling ratios, especially, r_{x}. It is suggested that the number of layer parallel to the XZ-plane increases.

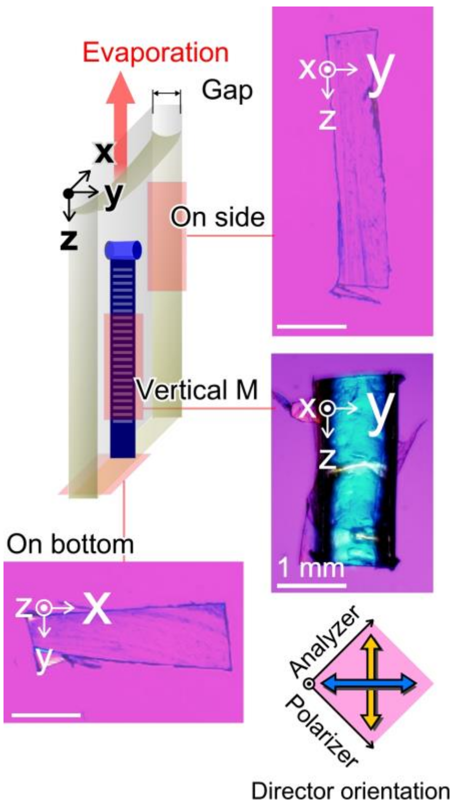

Figure S3. Polarized microscopic image of the chitosan casted film through crossed Nicols with a retardation plate (λ = 530 nm). The sample was prepared by drying a 2.5 wt% chitosan solution at 40 °C from a cell with a 1-mm gap.

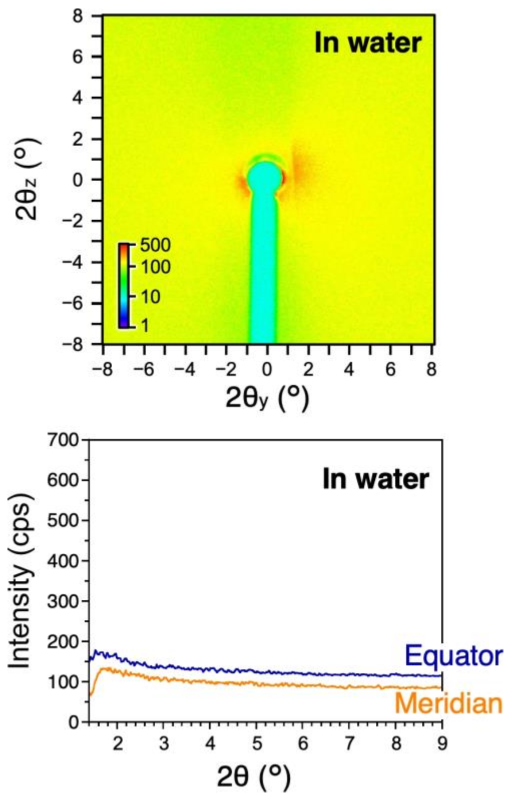

Figure S4. SAXS profiles of chitosan membrane under water environment. Room temperature: ~25 ℃.

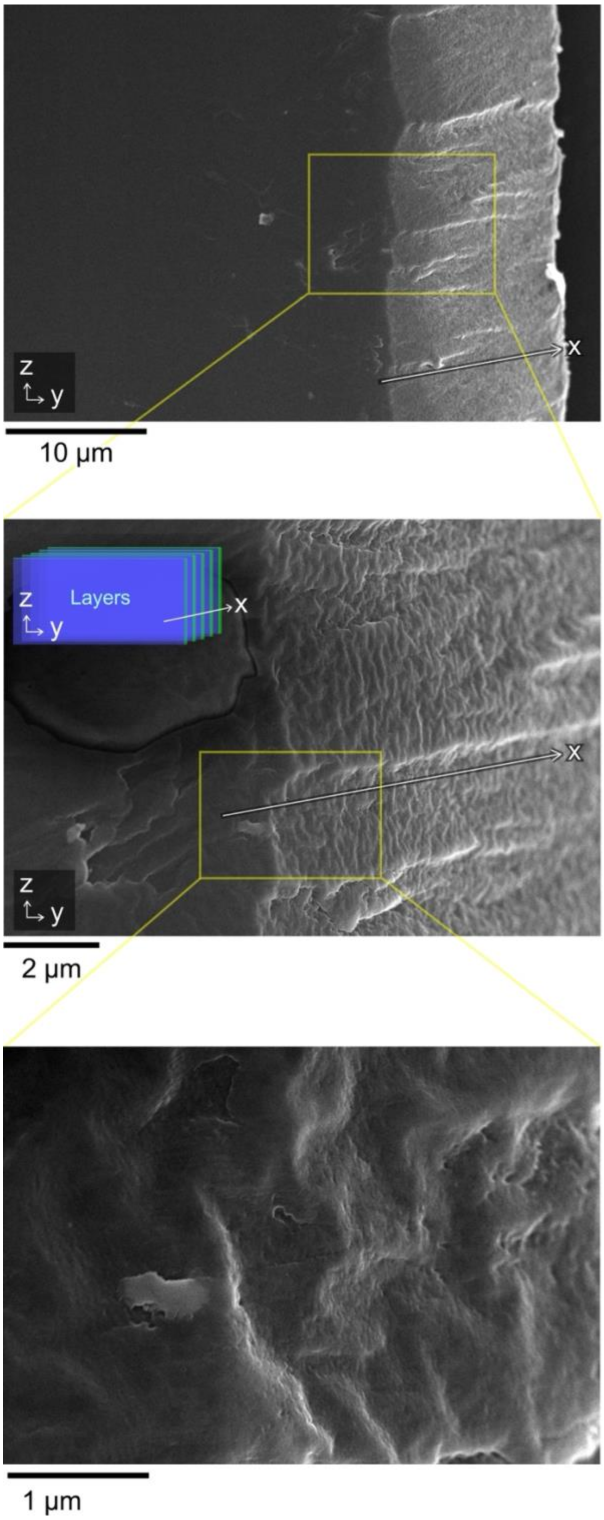

Figure S5. SEM images for the heat-dried sample prepared using the meniscus splitting method.

Table S1. Composition of pH buffer solution.

pH (21°C)	Citric acid 0.1 M	Na_2HPO_4 0.2 M
2.2	19.60 mL	0.40 mL
3.0	16.22 mL	3.78 mL
4.0	12.38 mL	7.62 mL
5.0	9.80 mL	10.20 mL
6.0	7.50 mL	12.50 mL
7.0	3.56 mL	16.44 mL
8.0	0.56 mL	19.44 mL

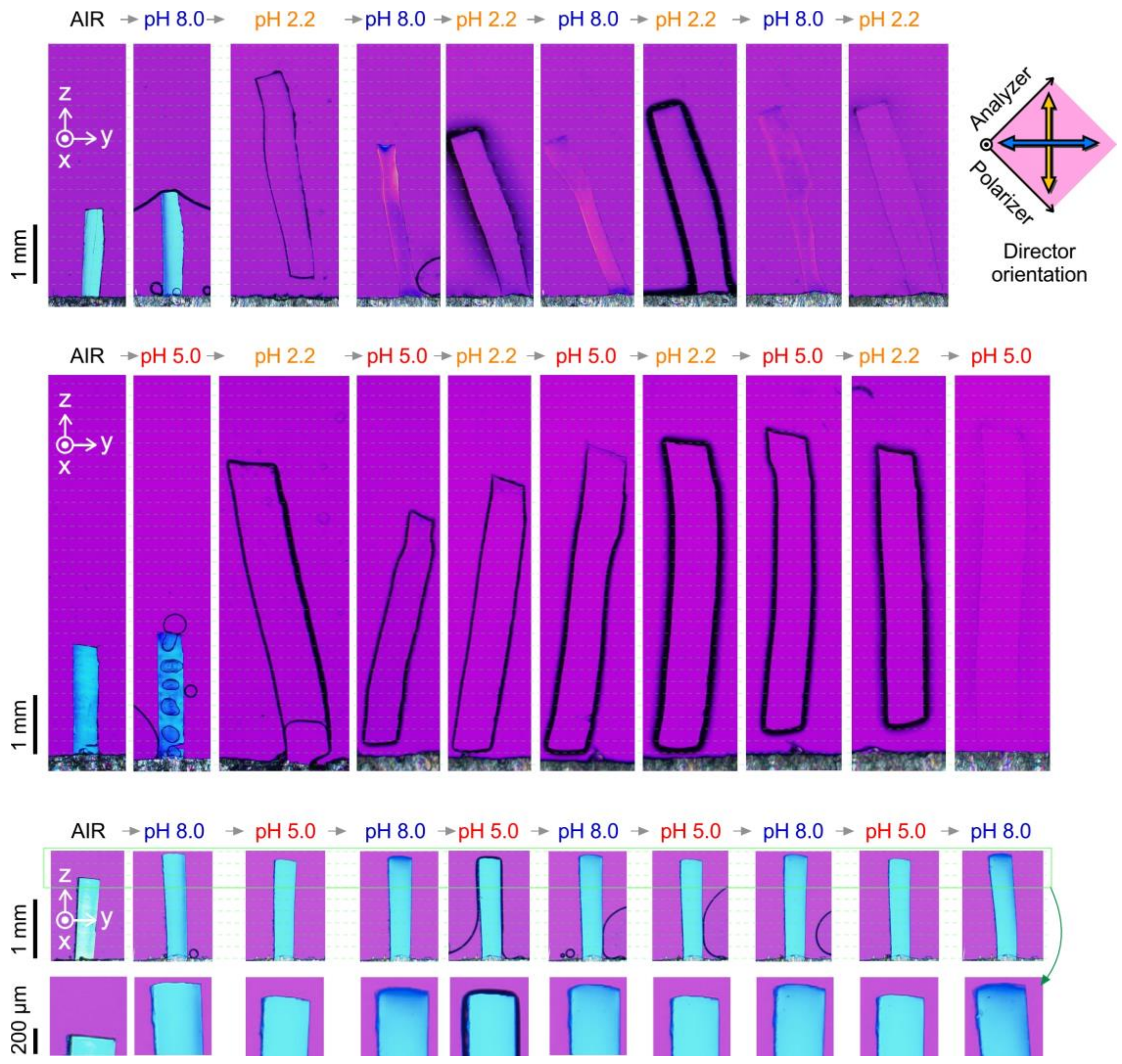

Figure S6. Polarized optical microscopic images of chitosan hydrogels during cyclic pH changes (pH 8 ⇄ pH 2.2, pH 5 ⇄ pH 2.2, and pH 8 ⇄ pH 5). Images were taken through crossed Nicols with a retardation plate (λ = 530 nm) in air and in aqueous buffer solutions at the given pH.
